# Supraglottic jet oxygenation and ventilation *via* nasopharyngeal airway for a patient with iatrogenic tracheoesophageal fistula: A case report

**DOI:** 10.3389/fmed.2023.1067424

**Published:** 2023-01-19

**Authors:** Yang Gu, Xiaowei Zhang, Keting Min, Juan Wei, Qing Zhou, Xin Lv, Ruowang Duan

**Affiliations:** ^1^Department of Anesthesiology, Shanghai Pulmonary Hospital, School of Medicine, Tongji University, Shanghai, China; ^2^Department of Anesthesiology, Shanghai Skin Disease Hospital, School of Medicine, Tongji University, Shanghai, China; ^3^Graduate School, Wannan Medical College, Wuhu, Anhui, China

**Keywords:** tracheoesophageal fistula, supraglottic jet oxygenation and ventilation, stent, nasopharyngeal airway, jet ventilation, rigid bronchoscope, flexible bronchoscope

## Abstract

**Background:**

Iatrogenic tracheoesophageal fistula (TEF) is a rare but life-threatening condition. No consensus has been reached regarding TEF treatment, though, stenting has been gaining popularity for less invasiveness than thoracic surgery. The airway management during stent placement for TEF could be challenging.

**Case presentations:**

We report a patient who suffered from TEF after cardiac surgery with symptoms of persistent coughing and aspiration. He who was admitted for stent placement but ended up in failure and referred to our institution for further treatment. We successfully took advantage of the supraglottic jet oxygenation and ventilation (SJOV) during stent placement.

**Conclusion:**

This is the first case so far describing SJOV in complicated stenting treatment. This demonstrates that SJOV can be applied for stent placement in TEF patients with restricted airways.

## Introduction

Tracheoesophageal fistula (TEF) is a rare iatrogenic late complication of tracheostomy in adults, with a prevalence of less than 1% (1). The main presentations include persistent cough, excessive secretions, recurrent aspiration, and gastric distention. Untreated TEF can deteriorate into acute respiratory distress syndrome and death (2). The treatment for this complication varies from stent deployment to thoracic surgical reconstruction. Ventilation management for iatrogenic TEF mainly relies on endotracheal tube (ET) and its modifications intubation (3, 4). However, the lack of high-level evidence has already prompted alternate measures (5). Herein, we present a rare case of successful supraglottic jet oxygenation and ventilation (SJOV)-assisted stent placement. Written informed consent and approval from the patient and research Ethics Committee of our hospital were obtained for medical education and publication.

## Case description

The patient was a 45-year-old man with a history of tracheostomy after surgery for hypertrophic cardiomyopathy, with a tracheostomy tube for 3 months. He was re-admitted to the same hospital because of coughing during the course of his diet and due to signs of pulmonary infection. The patient was diagnosed with TEF in the upper level after bronchoscopy examination and was scheduled to undergo tracheal stent placement after fasting and nasogastric tube insertion. However, the procedure failed because the stent was placed beneath the fistula and refractory to be adjusted appropriately or retracted, demanding transference. Additionally, his left upper limb experienced post-stroke paralysis. One day later, the patient was referred to our institution for further treatment.

**FIGURE 1:**
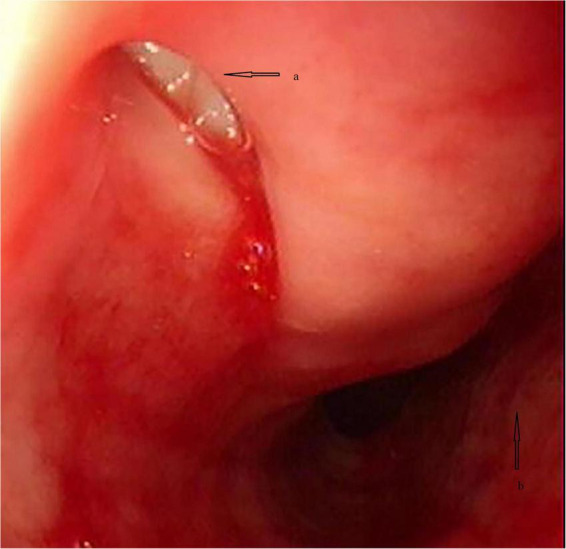
The pathological anatomy view of the trachea under bronchoscopy. **(a)** Fistula in the upper membranous wall; **(b)** the constricted trachea.

The plan was to adjust the implanted stent or replace it with a new one by the most experienced endoscopist in our institution. After standard monitoring and pre-oxygenation, rapid sequence induction was initiated, at his weight of 54 kg, with 2 mg midazolam, 20 μg sufentanil, 80 mg propofol, and 50 mg rocuronium, followed by a pumping infusion rate of 20 μg/h remifentanil and 220 mg/h propofol, as well as intermittent injections of 10 mg/30 min rocuronium. A rigid bronchoscope (RB) was inserted to examine the tracheal status, including the fistula and the stent, confirming that the fistula was in the upper membranous tracheal wall (Figure 1). The metal stent beneath the fistula had undergone deformity, and the trachea beneath the fistula was constricted (Figure 1). The endoscopist attempted to adjust the stent but failed, considering that the deformed stent might not be large enough to secure the fistula and expand the constricted part. Replacement with a larger covered self-expanding metal stent was justified.

**FIGURE 2:**
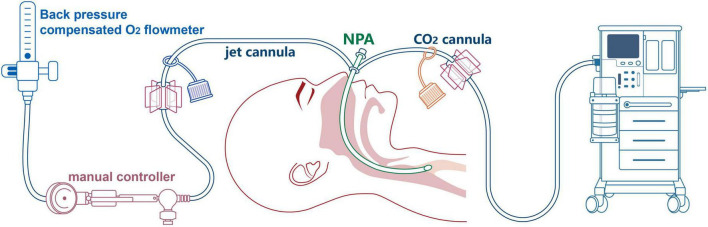
Pattern diagram of supraglottic jet oxygenation and ventilation (SJOV) *via* nasopharyngeal airway (NPA).

**FIGURE 3:**
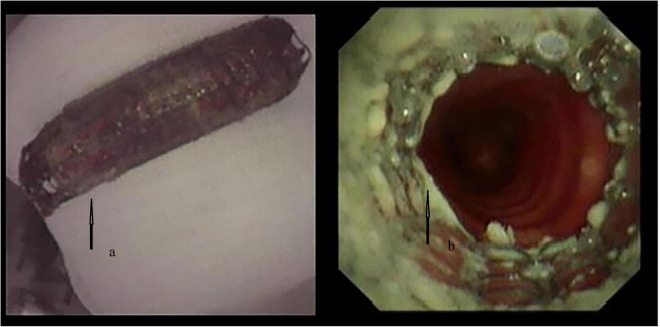
The deformed stent **(a)** and the covered metal stent mended the fistula and expanded the constricted trachea **(b)**.

High-flow oxygen (20 L/min) *via* the side port of the RB was used to counteract the leak; however, in less than 5 min, SpO_2_ deteriorated to 85%. Thus, RB was removed, and mask oxygenation was implemented to regain satisfactory oxygenation repeatedly until the entire stent was retracted by gentle extraction and twisting with biopsy forceps. As shown in the RB examination, the fistula lesion located in the upper membranous tracheal wall (approximately 2 cm beneath the glottis) precluded ET ventilation-assisted stent placement. Thus, we developed the idea of tubeless ventilation that we called SJOV (Figure 2). A nasopharyngeal airway (NPA) was inserted into the right nostril, with one of its catheters connected to a manual jet ventilator for oxygenation and ventilation, and the other was connected to an anesthesia machine for monitoring end-tidal carbon dioxide (ETCO_2_). The inserted length was the alae of the nose and the earlobe on the same side that was comparable to the length of the nostril to the retropharyngeal space. To ensure that the NPA was above the glottis, we used a video laryngoscope for adjustment. Ventilation was performed manually by an assistant anesthesiologist. Effective ventilation manifested as symmetrical chest rises and falls, clear breath sounds and no gurgling from the stomach. Oxygenation was well-maintained throughout the procedure with stable vital signs, and sufficient space was created for stent deployment guided by flexible bronchoscope (FB). A covered metal stent (18 × 60 mm) was successfully placed to mend the tracheal fistula and expand the constricted trachea (Figure 3). At the end of the procedure, arterial blood gas analysis showed acceptable ventilation efficacy (pH, 7.35; PaCO_2_: 47.9 mmHg). The patient was transferred to the post-anesthesia care unit after recovery of consciousness and regular spontaneous breathing.

## Discussion

This case describes a complicated ventilation strategy for stent treatment in a patient with iatrogenic TEF. Although stenting has been gaining popularity because it is less invasive, no consensus has been reached regarding TEF treatment. Considering the failed stent deployment and the medical history of the patient, the team placed it in high priority.

Aside from the constricted trachea, the previously stranded stent could have been incorporated into the mucosa and the granulation tissue originated from the tracheostomy (1), making it refractory to adjustment. During RB examination and stent retraction, desaturation occurred due to insufficient ventilation through the side port. High-flow oxygen ventilation could have been less compromised if no fistula in the trachea or oropharynx had been packed with gauze (6); or if the oxygen flow rate could be even higher (70 L/min) *via* a special ventilator like Optiflow^®^ (7), we might not have to do mask ventilation repeatedly. However, the dilemma lies in further injury due to the fistula with a less open airway system. Alternatively, if a much shorter time was needed by the endoscopist, we would have been able to tolerate the temporary hypoxia, but this was challenging, considering the cardiac surgery and stroke history of the patient. We thought that spontaneous breathing could have been maintained during RB examination, but with or without muscle relaxants in therapeutic RB showed no difference in safety parameters (including hypoxemia, respiratory failure, mortality et al.) (8); besides, a motionless state and still operating fields were more favorable to the endoscopist, which could be achieved with muscle relaxant; further, ventilation asynchrony could be saved with muscle relaxants. Total intravenous anesthesia was preferable in this case, as it had no leak compared to inhalation anesthesia.

Other ventilation strategies could have been applied during stent placement, including a de-cuffed modified ET, i-gel laryngeal mask airway (i-gel LMA), or classic laryngeal mask airway (LMA) ventilation with FB guidance (9, 10). ET was not suitable for this complicated stent deployment as was stated before. I-gel LMA could be an option with enough space left for stent placement, though, we were not equipped with. We could have achieved a successful ventilation with classic LMA along with FB with a working channel that allows the guidewire insertion. However, the working channel of FB will not allow passage of self-expanded metal stent. Sufficient space would likely to be created if a smaller tube passed through the glottis, which had been introduced in FB-assisted stenting by high-frequency jet ventilation (HFJV) with a 14F nylon insufflation catheter placed in the trachea (11). However, HFJV would be a hindrance to the endoscopist because the airway would be shared, and the procedure could misplace the ventilation tube. It could also be a risk factor for barotrauma in narrowing airway systems (12). With limited resources, therapeutic demands, and our concerns regarding the operational convenience, the hypoxia intolerance and airway protection, we believe that SJOV should be the most suitable.

Oxygenation was well-maintained throughout the manual SJOV without interfering with the procedure. However, this was not the case for ETCO_2_, although the CO_2_ monitoring catheter is valuable in fast procedures with fewer secretions (13), which was quite the opposite in our case. We were supposed to have transcutaneous capnography ready for this demanding non-invasive ventilation (NIV), which has been proven to be the best way to monitor NIV efficacy (14). Instead, we collaborated with the endoscopist by manual ventilation during SJOV, in which we could adjust from a larger tidal volume and higher frequency to asphyxia ventilation and cleared the secretions if necessary.

When SJOV was used in emergent situations with full stomach, the potential risks of aspiration increase significantly due to gastric distension resulted from possible air influx into the stomach and insufficient fasting time; on the other hand, however, the open airway system during SJOV also allows continuous forceful air outflows from the lungs which can be served as an aspiration preventive valve (15). Measures should be taken to prevent aspiration from happening: preoperative gastric tube insertion is still recommended; rapid sequence induction and proper Sellick’s maneuver with the patient in a 40° head-up position would be favorable (16); limited data (15, 17) showed that lowering the head position and setting mechanical ventilation frequency of over 80 per min or manual ventilation frequency of 20 per min with I:E ratio of 1:2 may prevent aspiration.

This is the first case report of SJOV in stent placement so far. It was less traumatic compared to conventional ET ventilation. Although, there are some limitations. First, ETCO_2_ monitoring was not applicable during the procedure, because CO_2_ sampling tube was easily clogged. Respiratory monitoring relied more on the observation of the chest movement and SpO_2_. Second, SJOV would have put the patient at the risk of aspiration if no gastric tube had been inserted.

Our strategy in this case was successful, proving that SJOV as an alternate ventilation strategy in stent placement is feasible and that communication with endoscopists is essential, especially when routine measures are inappropriate.

## Data availability statement

The raw data supporting the conclusions of this article will be made available by the authors, without undue reservation.

## Ethics statement

Written informed consent was obtained from the patient for the publication of any potentially identifiable images or data included in this article.

## Author contributions

YG and XZ contributed equally to the drafting and revision of the manuscript. KM, JW, and QZ revised the manuscript. RD and XL analyzed and interpreted the patient data, reviewed the literature, and revised the manuscript. All authors have approved the final manuscript.

## References

[1] Epstein S. Late complications of tracheostomy. *Respir Care.* (2005) 50:542–9. 15807919

[2] Shaida N Raj V Gopalan D. Iatrogenic tracheoesophageal fistula. *J Thoracic Oncol.* (2009) 4:1572. 10.1097/JTO.0b013e3181bbebee 20009912

[3] Chappell V Heck H Jr. Repair of large, iatrogenic, tracheo-esophageal fistulae. *Ann Thoracic Surg.* (2007) 83:705–6. 10.1016/j.athoracsur.2006.04.073 17258028

[4] Broemling N Campbell F. Anesthetic management of congenital tracheoesophageal fistula. *Paediatr Anaesth.* (2011) 21:1092–9. 10.1111/j.1460-9592.2010.03377.x 20723095

[5] Alslaim H Banooni A Shaltaf A Novotny N. Tracheoesophageal fistula in the developing world: are we ready for thoracoscopic repair? *Pediatr Surg Int.* (2020) 36:649–54. 10.1007/s00383-020-04639-7 32219560 PMC7223493

[6] Pathak V Welsby I Mahmood K Wahidi M MacIntyre N Shofer S. Ventilation and anesthetic approaches for rigid bronchoscopy. *Ann Am Thoracic Soc.* (2014) 11:628–34. 10.1513/AnnalsATS.201309-302FR 24635585

[7] Jung J Park J Lee M Chung Y. Apnoeic oxygenation using transnasal humidified rapid-insufflation ventilatory exchange during rigid bronchoscopy: a report of four cases. *J Int Med Res.* (2022) 50:3000605211068309. 10.1177/03000605211068309 35023372 PMC8785317

[8] Murgu S Laxmanan B Stoy S Egressy K Chaddha U Farooqui F Evaluation of safety and short-term outcomes of therapeutic rigid bronchoscopy using total intravenous anesthesia and spontaneous assisted ventilation. *Respiration.* (2020) 99:239–47. 10.1159/000504679 31851991

[9] Matsuda N Matsumoto S Nishimura T Wakamatsu H Kunihiro M Sakabe T. Perioperative management for placement of tracheobronchial stents. *J Anesth.* (2006) 20:113–7. 10.1007/s00540-005-0379-0 16633769

[10] Espinoza A Neumann K Halvorsen P Sundset A Kongerud J Fosse E. Critical airway obstruction: challenges in airway management and ventilation during therapeutic bronchoscopy. *J Bronchol Interv Pulmonol.* (2015) 22:41–7. 10.1097/lbr.0000000000000127 25590482

[11] Hautmann H Gamarra F Henke M Diehm S Huber R. High frequency jet ventilation in interventional fiberoptic bronchoscopy. *Anesth Anal.* (2000) 90:1436–40. 10.1097/00000539-200006000-00034 10825336

[12] Goudra B Singh P Borle A Farid N Harris K. Anesthesia for advanced bronchoscopic procedures: state-of-the-art review. *Lung.* (2015) 193:453–65. 10.1007/s00408-015-9733-7 25921014

[13] Zha B Wu Z Xie P Xiong H Xu L Wei H. Supraglottic jet oxygenation and ventilation reduces desaturation during bronchoscopy under moderate to deep sedation with propofol and remifentanil: a randomised controlled clinical trial. *Eur J Anaesthesiol.* (2021) 38:294–301. 10.1097/eja.0000000000001401 33234777 PMC7932749

[14] Georges M Rabec C Monin E Aho S Beltramo G Janssens J Monitoring of noninvasive ventilation: comparative analysis of different strategies. *Respir Res.* (2020) 21:324. 10.1186/s12931-020-01586-8 33302961 PMC7725884

[15] Jawan B Lee J. Aspiration in transtracheal jet ventilation. *Acta Anaesthesiol Scand.* (1996) 40:684–6. 10.1111/j.1399-6576.1996.tb04510.x 8836261

[16] Ovassapian A Salem M. Sellick’s maneuver: to do or not do. *Anesth Anal.* (2009) 109:1360–2. 10.1213/ANE.0b013e3181b763c0 19843769

[17] Yealy D Plewa M Reed J Kaplan R Ilkhanipour K Stewart R. Manual translaryngeal jet ventilation and the risk of aspiration in a canine model. *Ann Emerg Med.* (1990) 19:1238–41. 10.1016/s0196-0644(05)82280-2 2240717

